# Dislocation of total hip replacement in patients with fractures of the femoral neck: A prospective cohort study of 713 consecutive hips

**DOI:** 10.3109/17453670902930024

**Published:** 2009-04-01

**Authors:** Anders Enocson, Carl-Johan Hedbeck, Jan Tidermark, Hans Pettersson, Sari Ponzer, Lasse J Lapidus

**Affiliations:** ^1^Sections of Orthopedics, Department of Clinical Science and Education, Karolinska Institutet, Stockholm Söder HospitalStockholmSweden; ^2^Statistics, Department of Clinical Science and Education, Karolinska Institutet, Stockholm Söder HospitalStockholmSweden; ^3^Department of Orthopedics, Capio St. Göran’s HospitalStockholmSweden

## Abstract

**Background** Total hip replacement is increasingly used in active, relatively healthy elderly patients with fractures of the femoral neck. Dislocation of the prosthesis is a severe complication, and there is still controversy regarding the optimal surgical approach and its influence on stability. We analyzed factors influencing the stability of the total hip replacement, paying special attention to the surgical approach.

**Patients and methods** We included 713 consecutive hips in a series of 698 patients (573 females) who had undergone a primary total hip replacement (n = 311) for a non-pathological, displaced femoral neck fracture (Garden III or IV) or a secondary total hip replacement (n = 402) due to a fracture-healing complication after a femoral neck fracture. We used Cox regression to evaluate factors associated with prosthetic dislocation after the operation. Age, sex, indication for surgery, the surgeon’s experience, femoral head size, and surgical approach were tested as independent factors in the model.

**Results** The overall dislocation rate was 6%. The anterolateral surgical approach was associated with a lower risk of dislocation than the posterolateral approach with or without posterior repair (2%, 12%, and 14%, respectively (p < 0.001)). The posterolateral approach was the only factor associated with a significantly increased risk of dislocation, with a hazards ratio (HR) of 6 (2–14) for the posterolateral approach with posterior repair and of 6 (2–16) without posterior repair.

**Interpretation** In order to minimize the risk of dislocation, we recommend the use of the anterolateral approach for total hip replacement in patients with femoral neck fractures.

## Introduction

Different surgical methods are available for the treatment of displaced fractures of the femoral neck (Garden III and IV): internal fixation (IF), hemiarthroplasty (HA), and total hip replacement (THR). Despite the good results for THR reported in recent randomized controlled trials with regard to the need for revision surgery, hip function (Johansson et al. 2000, Tidermark et al. 2003, Keating et al. 2006), and health-related quality of life (HRQoL) (Tidermark et al. 2003, Blomfeldt et al. 2005, Keating et al. 2006), the proportion of patients treated with a THR in routine healthcare is not as high as would be expected (Bhandari et al. 2005). The risk of dislocation may be one major reason why orthopedic surgeons hesitate to perform a THR. Several studies have confirmed that the dislocation rate after a THR for a femoral neck fracture is considerably higher than what can be expected after a THR for osteoarthritis or rheumatoid arthritis (Woo and Morrey 1982, Berry et al. 2004, Meek et al. 2006). The principal surgical approaches for insertion of a THR are anterolateral (Hardinge 1982) or posterolateral (Moore 1957). The posterior approach can be performed with or without re-attachment of the short external rotators and/or the posterior joint capsule (posterior repair).

The influence of the surgical approach on stability is difficult to evaluate within the context of a conventional randomized controlled trial since most surgeons have their individual preferences regarding this issue. The best approach is probably randomization by surgeon, or a large prospective cohort trial in which the surgical approach used conforms to the preference of the treating surgeon.

We analyzed factors influencing the stability of a THR within the context of a large prospective cohort trial involving consecutive patients. We payed special attention to the surgical approach used.

## Patients and methods

The study was conducted at the Department of Orthopedics at Stockholm Söder Hospital between January 1, 1996, and December 31, 2005. This hospital is a public general hospital with a catchment area covering about 600,000 inhabitants. 713 consecutive hips that had undergone a THR in a series of 698 patients (573 females) were prospectively included in our clinical audit database. The patients were operated with a primary THR (n = 311) for a non-pathological displaced femoral neck fracture (Garden III or IV) or a secondary THR (n = 402) due to a fracture-healing complication (nonunion or avascular necrosis) after IF of a femoral neck fracture. Data on the type of prosthesis, including head size, indication for surgery (primary or secondary), surgical approach, level of surgeon’s experience (registrar or post-registrar/consultant), and reoperation were recorded. A 6- to 8-week prospective follow-up was performed within the context of a clinical audit. The patients were asked to report whether any complication had occurred after surgery and, if so, where it had been diagnosed and treated. Furthermore, all individual patient records were searched until December 31, 2006, or death, to find information about any dislocations and associated reoperations. Finally, the Swedish personal identification number was used to perform a search in the National Board of Health and Welfare’s national registry to find patients who had been treated elsewhere in Sweden for a dislocation up to December 31, 2006. Only 1 such case was found. The median follow-up time was 4.3 (0–11) years for all cases and 4.9 (1–11) years for those who were still alive on December 31, 2006.

The mean (SD; range) age was 78 (8.6; 46–96) years for women and 74 (9.8; 45–90) years for men. An anterolateral surgical approach was used on 463 hips and a posterolateral approach on 250 hips. A posterior repair with re-attachment of the short external rotators and/or the posterior joint capsule was performed in 110 of the 250 posterolateral approaches (Table 1). Postoperatively, the patients were mobilized bearing full weight, with the aid of crutches if needed. They were given instructions on how to avoid dislocation of the prosthesis and to abandon the crutches when feeling that it was safe to do so. There were no differences regarding age and sex comparing patients who were operated using the anterolateral or the posterolateral approach. However, the anterolateral approach was used significantly more often in primary THRs (p < 0.001), by inexperienced surgeons (p < 0.001), and in THRs with a 28-mm femoral head size (p < 0.001). The explanation for these variations was that the Charnley stem with a 22-mm head inserted by the posterolateral approach was used most often during the initial phase of the study. As a result of our own experience regarding surgical approach (Tidermark et al. 2003, Blomfeldt et al. 2007), we have gradually changed to the use of the anterolateral approach. However, at each time point, selection of the surgical approach was determined by the surgeon’s preference. There were 54 surgeons. The prostheses used were cemented (n = 707), uncemented (n = 5), and hybrid (n = 1) (Table 2).

**Table 1.** Baseline data for all patients included, with breakdown according to surgical approach

	All (n = 713)	Anterolateral (n = 463)	Posterolateral (n = 250)	p-value
Age, mean (SD)	76.8 (9.0)	77.2 (8.4)	76.1 (10.1)	0.1
Sex **^a^**				
Female	573 (80)	374 (81)	199 (80)	0.8
Male	140 (20)	89 (19)	51 (20)	
Indication **^a^**				
Primary	311 (44)	251 (54)	60 (24)	< 0.001
Secondary	402 (56)	212 (46)	190 (76)	
Surgeons’ experience **^a^**				
Registrar	77 (11)	64 (14)	13 (5)	< 0.001
Post-registrar	636 (89)	399 (86)	237 (95)	
Femoral head size **^a^**				
22-mm	171 (24)	14 (3)	157 (63)	< 0.001
28-mm	542 (76)	449 (97)	93 (37)	

**^a^** n (%)

**Table 2.** Number of prosthesis types and components used (n = 713)

	n (%)
Type of total hip arthroplasty	
Cemented	707 (99)
Uncemented	5 (<1)
Hybrid **^a^**	1 (<1)
Femoral component	
Cemented Exeter	538 (75)
Cemented Charnley	169 (24)
Uncemented BiMetric	6 (<1)
Acetabular component	
Cemented Exeter	537 (75)
Cemented Charnley	171 (24)
Uncemented Trilogy	3 (<1)
Uncemented Romanus	2 (<1)

**^a^** Cemented femoral and uncemented acetabular components

During the study period, we did not routinely use any validated instrument for assessment of cognitive function. However, information regarding any diagnosed dementia was available.

The study was conducted in conformity with the Helsinki Declaration and was approved by the local Ethics Committee in Stockholm (number 2006/1409-31/4 and 2007/1309-32).

### Statistics

The Mann-Whitney U-test was used for scale variables in independent groups. Nominal variables were tested by the Chi-squared test or Fisher’s exact test. All tests were two-sided. We used Cox regression to evaluate factors associated with prosthetic dislocation after the operation. Age, sex, indication for surgery, the surgeon’s experience, femoral head size, and surgical approach were tested as independent factors in the model. First, crude associations for each factor were studied in univariable models. Secondly, a multivariable model with all independent factors was used to study the adjusted associations. The associations are presented as hazards ratios (HRs) with 95% confidence intervals (CIs). The associations were tested using the Wald test and they were considered significant if p < 0.05. All tests were two-sided. The statistical software used was SPSS version 15.0 for Windows.

## Results

Dislocation of the THR occurred in 41 of the 713 hips, giving an overall dislocation rate of 6%. The anterolateral surgical approach was associated with a lower risk of dislocation than the posterolateral approach with or without posterior repair (2%, 12%, and 14%, respectively) (p < 0.001). The univariate analysis indicated a significantly increased risk of dislocations in hips operated upon using the posterolateral approach with or without posterior repair, and for hips with the 22-mm femoral head (Table 3). However, the multivariable Cox regression analysis showed that the posterolateral approach was the only factor associated with a significantly increased risk of dislocation, with HR of 6 (2–14) for the posterolateral approach with posterior repair and 6 (2–16) for the posterolateral approach without posterior repair. The patient’s age, sex, the indication for surgery, the experience of the surgeon, and the femoral head size had no influence on the dislocation rate.

**Table 3.** Cox regression to evaluate factors associated with prosthetic dislocation (n = 713)

Explanatory	n	Dislocation rate (%)	Univariate		Multivariate	
			HR **^a^** (95% CI)	p-value	HR **^a^** (95% CI)	p-value
Age						
< 78 years	349	6.6	1 ^**b**^		1 ^**b**^	
≥ 78 years	364	4.9	0.8 (0.4–1.4)	0.4	0.8 (0.4–1.5)	0.4
Sex						
Male	140	5.9	1 ^**b**^		1 ^**b**^	
Female	573	5.0	1.2 (0.5–2.6)	0.7	1.2 (0.5–2.7)	0.7
Indication						
Primary	311	4.5	1 ^**b**^		1 ^**b**^	
Secondary	402	6.7	1.5 (0.8–2.8)	0.2	0.8 (0.4–1.6)	0.5
Surgeon’s experience						
Registrar	77	3.9	1 ^**b**^		1 ^**b**^	
Post-registrar	636	6.0	1.4 (0.4–4.5)	0.6	0.9 (0.3–2.8	0.8
Femoral head size						
22-mm	171	13.5	1 ^**b**^		1 ^**b**^	
28-mm	542	3.3	0.3 (0.1–0.5)	< 0.001	0.7 (0.3–1.5)	0.4
Surgical approach **^c^**						
A-L	463	1.9	1 ^**b**^		1 ^**b**^	
P-L with posterior repair	110	11.8	6.1 (2.6–14)	< 0.001	5.5 (2.1–14)	< 0.001
P-L without posterior repair	140	13.6	6.8 (3.1–15)	< 0.001	5.7 (2.0–16)	0.001

**^a^** HR: hazards ratio.

**^b^** Reference

**^c^** A-L: anterolateral; P-L: posterolateral.

There was no selection bias of patients with dementia to any of the surgical approaches. Of the 463 patients who were operated on using the anterolateral approach, 18 (4%) had a diagnosis of dementia as compared to 6 of 250 (2%) who were operated on using the posterolateral approach (p = 0.4).

The first dislocation occurred early (within 6 weeks) in 24 of the 41 patients with dislocation. Closed reduction was successful for 39 of these 41 patients. One of the remaining 2 patients (posterolateral approach) was reoperated with a socket wall augmentation device and had no further dislocations. The other patient (anterolateral approach) underwent open reduction, developed a deep infection, and had the prosthesis extracted. 25 of the 39 patients who were initially successfully treated with closed reduction had recurrent dislocations: 6 of the 8 patients operated on using the anterolateral approach and 19 of the 31 patients treated using the posterolateral approach (p = 0.7). Revision surgery due to instability was performed on 11 of the 41 patients (including the 2 patients treated with a primary open procedure) during the study period: 3 of 9 patients operated on using the anterolateral approach, and 8 of 32 patients treated using the posterolateral approach (p = 0.7).

When comparing patients operated on using the anterolateral approach with those operated on using the posterolateral approach, there were no differences regarding nerve injuries, deep infections, or mortality within the first year after surgery. Revision surgery for reasons other than dislocation and general complications within the first 6 weeks such as pneumonia, or cardiovascular, thromboembolic, or cerebrovascular events were also equally distributed between the 2 groups (data not shown).

## Discussion

The dislocation rate of 2% after the anterolateral approach in this study is similar to that reported for THR using the anterolateral approach in two randomized controlled trials (RCTs) from our institution. Tidermark et al. (2003) reported a 2% dislocation rate after THR in an RCT comparing IF and THR, and Blomfeldt et al. (2007) had no dislocations in any of the arthroplasty groups in an RCT comparing bipolar HA and THR. These figures are similar to the 1% dislocation rate reported for all arthroplasties in the multicenter RCT by Keating et al. (2006), comparing IF, bipolar HA, and THR. A higher dislocation rate (8%) was reported by Baker et al. (2006) in an RCT comparing IF with THR, also using the anterolateral approach.

The significantly higher dislocation rates after the posterolateral approach with and without posterior repair (12% and 14%, respectively) were of the same magnitude as those reported for THR groups in RCTs using the posterolateral approach. Skinner and co-workers (1989) reported a 13% dislocation rate for THR in an RCT comparing IF, unipolar HA, and THR. In a 13-year follow-up of the same patient population, the dislocation rate in the THR group had increased to 20% (Ravikumar and Marsh 2000). This cumulative long-term risk of dislocation has been highlighted in other recent studies (von Knoch et al. 2002, Berry et al. 2004). In another RCT comparing IF and THR, Johansson et al. (2000) reported a dislocation rate of 22% after THR. An interesting additional finding in that study was that the dislocation rate was elevated in patients with mental dysfunction: 32%, as compared to 12% in lucid patients.This supports the notion that patients with severe cognitive dysfunction who—besides their increased risk of dislocation—also have a worse outcome regarding function and mortality (Söderqvist et al. 2006) should not be considered for the THR procedure.

Two-thirds of the patients had at least one recurrent dislocation after the first closed reduction. Furthermore, revision surgery due to instability was performed in 11 of 41 of the patients during the study period, which was slightly lower than the 35% reported by Woo and Morrey (1982) in a study with a similar follow-up time. These high figures underscore the fact that instability is a severe complication, often necessitating major revision surgery in order to regain stability—a procedure that has far from always been successful. Woo and Morrey (1982) reported that the instability persisted in one-third of the hips revised due to recurrent dislocations.

Repair of the posterior structures, i.e. the short external rotators and/or the posterior joint capsule, has been reported to increase stability after a posterolateral approach. In a recent meta-analysis by Kwon et al. (2006) comprising 4,115 patients from 5 studies, the dislocation rate for THR was 0.5% for patients with a posterior repair and 5% for those without. However, the conclusion that a posterior repair greatly reduces the risk of dislocation is probably most valid for patients with degenerative joint disease. Only 2 of the studies included reported on the preoperative diagnosis and, in those, only a minority of the patients had had fractures of the femoral neck or had sequelae after femoral neck fractures (5% and 15%, respectively).

We have recently analyzed factors influencing the stability of a hemiarthroplasty (HA) in patients with femoral neck fractures, with special reference to the surgical approach and within the context of a prospective cohort trial (Enocson et al. 2008). Although the patients selected for HA were generally older (84 years) and less active than patients selected for THR, comparisons between the two studies are of interest. In that study the anterolateral approach was associated with a lower risk of dislocation than the posterolateral approach, with or without posterior repair (3%, 9%, and 13%, respectively) (p?<?0.001). The multivariate regression analysis showed that the posterolateral approach was the only factor associated with an increased risk of dislocation and the results also showed a trend towards improved stability with a posterior repair. The positive effect of a posterior repair in patients treated with an HA could not be confirmed in the present study on THR.

Larger femoral head size has been suggested to reduce the risk of dislocation. This has been reported in clinical studies (Hedlundh et al. 1996a, Amstutz et al. 2004, Berry et al. 2005) as well as in experimental ones (Kluess et al. 2007), whereas some studies have not demonstrated this positive effect (Woo and Morrey 1982). Our univariate regression analysis suggested a lower risk of dislocation with the 28-mm head than with the 22-mm head. This finding could, however, be explained by the fact that the majority of the patients with a 22-mm femoral head were operated using a posterolateral approach, and the finding could not be verified in the multivariable analysis. Perhaps the size of the head must be over 28 mm in order to improve the stability (Hedlundh et al. 1996a). On the other hand, most of these fracture patients are females and in a considerable number of patients we have used an acetabular component with an outside diameter of 40 mm. Increasing the femoral head size to 32 mm or more might reduce the thickness of the polyethylene to a critical level and could thereby jeopardize the long-term outcome.

We did not find any difference in dislocation rate between primary and secondary THRs, which is in contrast to the results of some previous studies. In a prospective case-control study, McKinley and Robinson (2002) reported an increased rate of dislocations after a secondary THR (20%) compared to a primary one (8%), all of which were performed via a posterior approach. A similar finding was reported by Woo and Morrey (1982): 12% after a secondary THR and 9% after a primary one. There are no obvious reasons why a secondary THR should have an increased dislocation rate. The surgical procedure during a secondary THR is often more technically demanding. In addition, these patients have often suffered pain and disability for a long time before the secondary THR, probably resulting in poor muscle function. On the other hand, the secondary THR is usually an elective procedure with an optimized patient. Moreover, the stiff joint capsule developed during the (often) long time to failure of the internal fixation may also reduce the risk of instability, comparable to that of patients with a degenerative joint disease.

It has been reported that inexperienced surgeons are associated with a higher incidence of dislocation than more experienced surgeons (Hedlundh et al. 1996b). We could not confirm this finding, which may be due to the fact that the routine at our department requires that an inexperienced surgeon is always assisted by a more experienced one.

One limitation of our study was the lack of a preoperative assessment of cognitive function based on a validated instrument. Cognitive dysfunction seems to be a substantial risk factor for dislocation in hip fracture patients treated with a THR (Johansson et al. 2000) and for a long time we have avoided performing THR in patients with severe cognitive dysfunction/dementia. Only 3% of our patients had a diagnosis of dementia, and there did not seem to be any selection bias with regard to dementia for any of the approaches. However, performance of a THR may be necessary in individual patients with severe cognitive dysfunction/dementia, e.g. in patients with severe pain due to avascular necrosis.

Another limitation of our study was that the position of the implant was not assessed. Theoretically, the higher dislocation rate after the posterolateral approach may (apart from the soft tissue injury) be partly a result of a higher frequency of poorly positioned implants. However, the possibility for optimal implant position is an important characteristic of the surgical approach.

The strengths of our study were the large number of consecutively entered patients, the relatively long follow-up time, and the validation of dislocation data via the nationwide registry of the Swedish National Board of Health and Welfare. Since this particular issue is difficult to address within the context of a conventional randomized study, a large prospective cohort trial such as the present one, including consecutive patients and in which the selection of the surgical approach at each point in time was determined is the individual surgeon’s preference, is a good approach—one that is only surpassed in quality by a trial using randomization by surgeon. Thus, we have good reason to assume that our conclusions regarding the risk factors for dislocation that we studied are valid for this patient cohort.

In summary, a dislocation after a THR in patients with a femoral neck fracture is a relatively common, severe, and expensive complication (Meek et al. 2006, Sanchez-Sotelo et al. 2006). If we can avoid dislocations and deep infections in this patient group, the lifetime risk of undergoing revision surgery is very low (The Swedish National Hip Arthoplasty Registry 2008).

## References

[CIT0001] Amstutz HC, Le Duff MJ, Beaule PE (2004). Prevention and treatment of dislocation after total hip replacement using large diameter balls.. Clin Orthop.

[CIT0002] Baker RP, Squires B, Gargan MF, Bannister GC (2006). Total hip arthroplasty and hemiarthroplasty in mobile, independent patients with a displaced intracapsular fracture of the femoral neck. A randomized, controlled trial.. J Bone Joint Surg (Am).

[CIT0003] Berry DJ, von Knoch M (2004). The cumulative long-term risk of dislocation after primary Charnley total hip arthroplasty.. J Bone Joint Surg (Am).

[CIT0004] Berry DJ, von Knoch M (2005). Effect of femoral head diameter and operative approach on risk of dislocation after primary total hip arthroplasty.. J Bone Joint Surg (Am).

[CIT0005] Bhandari M, Devereaux PJ, Tornetta P, Swiontkowski MF, Berry DJ, Haidukewych G, Schemitsch EH, Hanson BP, Koval K, Dirschl D, Leece P, Keel M, Petrisor B, Heetveld M, Guyatt GH (2005). Operative management of displaced femoral neck fractures in elderly patients. An international survey.. J Bone Joint Surg (Am).

[CIT0006] Blomfeldt R, Törnkvist H, Ponzer S, Söderqvist A, Tidermark J (2005). Comparison of internal fixation with total hip replacement for displaced femoral neck fractures. Randomized, controlled trial performed at four years.. J Bone Joint Surg (Am).

[CIT0007] Blomfeldt R, Törnkvist H, Eriksson K, Söderqvist A, Ponzer S, Tidermark J (2007). A randomised controlled trial comparing bipolar hemiarthroplasty with total hip replacement for displaced intracapsular fractures of the femoral neck in elderly patients.. J Bone Joint Surg (Br).

[CIT0008] Enocson A, Tidermark J, Törnkvist H, Lapidus LJ (2008). Dislocation of hemiarthroplasty after femoral neck fracture: better outcome after the anterolateral approach in a prospective cohort study on 739 consecutive hips.. Acta Orthop.

[CIT0009] Hardinge K (1982). The direct lateral approach to the hip.. J Bone Joint Surg (Br).

[CIT0010] Hedlundh U, Ahnfelt L, Hybbinette CH, Wallinder L, Weckström J, Fredin H (1996a). Dislocations and the femoral head size in primary total hip arthroplasty.. Clin Orthop.

[CIT0011] Hedlundh U, Ahnfelt L, Hybbinette CH, Weckström J, Fredin H (1996b). Surgical experience related to dislocations after total hip arthroplasty.. J Bone Joint Surg (Br).

[CIT0012] Johansson T, Jacobsson SA, Ivarsson I, Knutsson A, Wahlström O (2000). Internal fixation versus total hip arthroplasty in the treatment of displaced femoral neck fractures: a prospective randomized study of 100 hips.. Acta Orthop Scand.

[CIT0013] Keating JF, Grant A, Masson M, Scott NW, Forbes JF (2006). Randomized comparison of reduction and fixation, bipolar hemiarthroplasty, and total hip arthroplasty. Treatment of displaced intracapsular hip fractures in healthy older patients.. J Bone Joint Surg (Am).

[CIT0014] Kluess D, Martin H, Mittelmeier W, Schmitz KP, Bader R (2007). Influence of femoral head size on impingement, dislocation and stress distribution in total hip replacement.. Med Eng Phys.

[CIT0015] Kwon MS, Kuskowski M, Mulhall KJ, Macaulay W, Brown TE, Saleh KJ (2006). Does surgical approach affect total hip arthroplasty dislocation rates?. Clin Orthop.

[CIT0016] Mckinley JC, Robinson CM (2002). Treatment of displaced intracapsular hip fractures with total hip arthroplasty: comparison of primary arthroplasty with early salvage arthroplasty after failed internal fixation.. J Bone Joint Surg (Am).

[CIT0017] Meek RM, Allan DB, Mcphillips G, Kerr L, Howie CR (2006). Epidemiology of dislocation after total hip arthroplasty.. Clin Orthop.

[CIT0018] Moore AT (1957). The self-locking metal hip prosthesis.. J Bone Joint Surg (Am).

[CIT0019] Ravikumar KJ, Marsh G (2000). Internal fixation versus hemiarthroplasty versus total hip arthroplasty for displaced subcapital fractures of femur-13 year results of a prospective randomised study.. Injury.

[CIT0020] Sanchez-sotelo J, Haidukewych GJ, Boberg CJ (2006). Hospital cost of dislocation after primary total hip arthroplasty.. J Bone Joint Surg (Am).

[CIT0021] Skinner P, Riley D, Ellery J, Beaumont A, Coumine R, Shafighian B (1989). Displaced subcapital fractures of the femur: a prospective randomized comparison of internal fixation, hemiarthroplasty and total hip replacement.. Injury.

[CIT0022] Söderqvist A, Miedel R, Ponzer S, Tidermark J (2006). The influence of cognitive function on outcome after a hip fracture.. J Bone Joint Surg (Am).

[CIT0023] Tidermark J, Ponzer S, Svensson O, Söderqvist A, Törnkvist H (2003). Internal fixation compared with total hip replacement for displaced femoral neck fractures in the elderly. A randomised, controlled trial.. J Bone Joint Surg (Br).

[CIT0024] von Knoch M, Berry DJ, Harmsen WS, Morrey BF (2002). Late dislocation after total hip arthroplasty.. J Bone Joint Surg (Am).

[CIT0025] Woo RY, Morrey BF (1982). Dislocations after total hip arthroplasty.. J Bone Joint Surg (Am).

